# Metabolic syndrome increases osteoarthritis risk: findings from the UK Biobank prospective cohort study

**DOI:** 10.1186/s12889-024-17682-z

**Published:** 2024-01-19

**Authors:** Shiyong Zhang, Danni Wang, Jinyu Zhao, Haitong Zhao, Peng Xie, Linli Zheng, Puyi Sheng, Jinqiu Yuan, Bin Xia, Fuxin Wei, Ziji Zhang

**Affiliations:** 1 Department of Joint Surgery, the First Affiliated Hospital, Sun Yat-Sen University, Guangzhou, 510080 Guangdong China; 2 Department of Epidemiology and Biostatistics, Clinical Big Data Research Center, The Seventh Affiliated Hospital, Sun Yat-Sen University, Shenzhen, 518000 Guangdong China; 3 Chinese Health RIsk MAnagement Collaboration (CHRIMAC), Shenzhen, 518000 Guangdong China; 4 Guangdong Provincial Key Laboratory of Gastroenterology, Center for Digestive Disease, The Seventh Affiliated Hospital, Sun Yat-Sen University, Shenzhen, 518000 Guangdong China; 5 Department of Orthopedics, the Seventh Affiliated Hospital, Sun Yat-Sen University, Shenzhen, 518000 Guangdong China; 6 Department of General Surgery, The First Hospital of Lanzhou University, Lanzhou, 730000 Gansu China; 7 Evidence Based Social Science Research Center, School of Public Health, Lanzhou University, Lanzhou, 730000 Gansu China; 8 Digestive Diseases Center, The Seventh Affiliated Hospital,, Sun Yat-Sen University, Shenzhen, 518107 Guangdong China; 9 Guangdong Provincial Key Laboratory of Digestive Cancer Research, The Seventh Affiliated Hospital of Sun Yat-Sen University, No. 628 Zhenyuan Road, Shenzhen, 518107 Guangdong China

**Keywords:** Metabolic syndrome (MetS), Osteoarthritis (OA), C-reactive protein (CRP), UK Biobank

## Abstract

**Objective:**

The association between Metabolic Syndrome (MetS), its components, and the risk of osteoarthritis (OA) has been a topic of conflicting evidence in different studies. The aim of this present study is to investigate the association between MetS, its components, and the risk of OA using data from the UK Biobank.

**Methods:**

A prospective cohort study was conducted in the UK Biobank to assess the risk of osteoarthritis (OA) related to MetS. MetS was defined according to the criteria set by the International Diabetes Federation (IDF). Additionally, lifestyle factors, medications, and the inflammatory marker C-reactive protein (CRP) were included in the model. Cox proportional hazards regression was used to calculate hazard ratios (HR) and 95% confidence intervals (CI). The cumulative risk of OA was analyzed using Kaplan–Meier curves and log-rank tests. To explore potential nonlinear associations between MetS components and OA risk, a restricted cubic splines (RCS) model was employed. In addition, the polygenic risk score (PRS) of OA was calculated to characterize individual genetic risk.

**Results:**

A total of 45,581 cases of OA were identified among 370,311 participants, with a median follow-up time of 12.48 years. The study found that individuals with MetS had a 15% higher risk of developing OA (HR = 1.15, 95%CI:1.12–1.19). Additionally, central obesity was associated with a 58% increased risk of OA (HR = 1.58, 95%CI:1.5–1.66), while hyperglycemia was linked to a 13% higher risk (HR = 1.13, 95%CI:1.1–1.15). Dyslipidemia, specifically in triglycerides (HR = 1.07, 95%CI:1.05–1.09) and high-density lipoprotein (HR = 1.05, 95%CI:1.02–1.07), was also found to be slightly associated with OA risk. When stratified by PRS, those in the high PRS group had a significantly higher risk of OA compared to those with a low PRS, whereas no interaction was found between MetS and PRS on OA risks. Furthermore, the presence of MetS significantly increased the risk of OA by up to 35% in individuals with elevated CRP levels (HR = 1.35, 95% CI:1.3–1.4).

**Conclusion:**

MetS and its components have been found to be associated with an increased risk of OA, particularly in individuals with elevated levels of CRP. These findings highlight the significance of managing MetS as a preventive and intervention measure for OA.

**Supplementary Information:**

The online version contains supplementary material available at 10.1186/s12889-024-17682-z.

## Introduction

Osteoarthritis (OA) is the most common chronic degenerative joint disorder, with symptoms including joint pain, deformity, and limited range of motion [1]. The incidence of OA is on the rise due to modern lifestyles and population ageing. It is estimated that there are more than 250 million OA patients worldwide [2]. According to the economic burden studies of OA, the per capita disease management cost for OA patients worldwide is $700-$15,600, which imposes an enormous economic burden on health systems [3, 4]. Although OA can affect any joint in the body, it usually seems more common in the hip and knee [5]. Therefore, OA was once considered a result of "wear and tear" due to excessive movement and loading of the joints. However, growing evidence suggests that the development and progression of OA should be attributed to a combination of factors, including chronic low-grade inflammation, obesity, hyperglycemia, and unhealthy lifestyles [6–8]. Considering this, a new phenotype of OA, termed "Metabolic osteoarthritis" or "Metabolic syndrome-associated osteoarthritis", is beginning to receive more attention [9, 10].

Metabolic syndrome (MetS) is not the name of a specific disease but rather an ensemble of clinical risk factors, including central obesity, hyperglycemia/insulin resistance, elevated blood pressure, and disorders of lipid metabolism [11]. The International Diabetes Federation (IDF) define MetS as a clinical syndrome characterized by obesity, hyperlipidemia, reduced high-density lipoprotein (HDL), hypertension, and hyperglycemia. MetS and its components have now been identified to be associated with a variety of diseases, such as inflammatory diseases, bladder cancer, and pancreatic cancer [12–14]. Modern lifestyles, such as smoking, alcohol consumption and sedentary behaviors, are also thought to play a role in the development of obesity, MetS and OA [15]. Previous studies have explored the potential correlation between MetS and OA risk, but the evidence is contradictory. Jansen et al. found in a prospective cohort study based on 682 women that the MetS and its components including waist circumference and HDL cholesterol levels were associated with OA progression, even after adjustment for baseline BMI [10]. However, Niu et al. found in a prospective study based on 991 Framingham Study participants that the MetS and its components were associated with incident OA, but these associations became nonsignificant after adjusting for BMI, with the exception of hypertension, which was consistently associated with OA [16]. We now appreciate that low-grade systemic inflammation has an important role in the progression of OA [17].

Association of C-reactive protein (CRP), the most commonly used inflammatory marker, with OA has been widely studied, but the evidence is conflicting [18, 19]. Meanwhile, the association of MetS with CRP is complex. The MetS component, such as obesity, can promote inflammation and elevate CRP by secreting pro-inflammatory adipokines; also, CRP can increase insulin resistance and hyperglycemia by interfering with insulin signalling [20–22]. In a prospective study based on 5171 participants, MetS was found to be associated with an increased incidence of knee OA, but this association was mainly contributed by BMI. Meanwhile, CRP was not associated with the occurrence of either hip or knee OA. However, the authors acknowledged a potential source of bias in the study, as it relied on arthroplasty-defined OA as an outcome, possibly leading to selection bias in favor of healthier patients [23].

Both MetS and CRP have been presented to be potentially associated with an increased risk of OA [23, 24]. although the association between MetS and OA risk has been explored in more studies, this association has not been established in low-grade inflammatory states. The present study aimed to investigate the relationship between MetS and its components and the risk of OA, considering both linear and nonlinear associations. Additionally, a joint analysis of MetS, CRP, and OA risk was conducted to determine if this association remains significant in the presence of inflammation.

## Materials and methods

### Participants and data access

We used the UK Biobank database as the data source (application number 51671, approved August 2019). The UK Biobank is a large prospective cohort study encompassing over 500,000 participants and has now provided reliable population data for numerous epidemiological studies since the health information of participants was collected in 2006 [25, 26]. All participants in the UK Biobank provided informed written consent at the time of inclusion in the cohort, and all information was available for scientific research. We first selected a cohort of a total of 501,109 participants aged 37–73 years (female: 272,632; male: 228,477). At baseline, we excluded participants who lacked any metabolic composition data (*n* = 74,697) and those who were lost to follow-up (*n* = 1,297). Additionally, individuals with a diagnosis of OA at any site or a self-reported history of OA were excluded (*n* = 54,804). Finally, a total of 370,311 participants (female: 195,700; male: 174,611) were included in this study. Baseline characteristics, including demographic information, metabolic profiles, and other relevant data, can be found in Table 1.

**Table 1:** Baseline characteristics of participants stratified by MetS in the UK biobank cohort

**Characteristics**	**MetS**		**Overall** ***N*** = 370,311
	**No** (*N* = 281,018)	**Yes** (*N* = 89,293)	
Mean (SD) age, years	55.97 (8.18)	57.96 (7.78)	56.45 (8.13)
**Gender**			
Male, N (%)	129,116(45.9)	45,495(51.0)	174,611(47.2)
Female, N (%)	151,902(54.1)	43,798(49.0)	195,700(52.8)
**Ethnic, (white) N (%)**	265,662 (94.5)	84,292 (94.4)	349,954 (94.5)
**Age, years, N (%)**			
< 55	133,964 (47.7)	33,162 (37.1)	167,126 (45.1)
55–65	112,849 (40.2)	41,462 (46.4)	154,311 (41.7)
> 65	34,205 (12.2)	14,669 (16.4)	48,874 (13.2)
Mean (SD) IMD	16.32 (13.39)	19.12 (14.99)	17.00 (13.84)
Mean (SD) HDL, mmol/L	1.52 (0.38)	1.23 (0.29)	1.45 (0.38)
Mean (SD) TG, mmol/L	1.52 (0.86)	2.39 (1.17)	1.73 (1.01)
Mean (SD) fasting glucose, mmol/L	4.97 (0.93)	5.74 (1.81)	5.11 (1.23)
Mean (SD) SBP, mmHg	137.33 (19.67)	145.98 (18.15)	139.42 (19.67)
Mean (SD) DBP, mmHg	80.98 (10.56)	86.28 (10.30)	82.26 (10.74)
Waist circumference (cm)	85.44 (10.76)	103.78 (10.55)	89.86 (13.28)
Mean (SD) BMI, kg/m^2^	25.60 (3.47)	32.16 (4.29)	27.18 (4.63)
**Alcohol consumption, N (%)**			
Daily or almost daily	60,798 (21.6)	15,557 (17.4)	76,355 (20.6)
1–4 times a week	143,164 (50.9)	40,513 (45.4)	183,677 (49.6)
1–3 times a month	29,825 (10.6)	11,316 (12.7)	41,141 (11.1)
Special occasions only/Never	47,231 (16.8)	21,907 (24.5)	69,138 (18.7)
Median (IQR) Physical activity, MET hours/week	31.54(46.48)	23.44(41.02)	29.55(45.55)
**Smoking status(%)**			
Current	29,504 (10.5)	9,865 (11.0)	39,369 (10.6)
Previous	90,020 (32.0)	35,033 (39.2)	125,053 (33.8)
Never	161,494 (57.5)	44,395 (49.7)	205,889 (55.6)
NSAIDs, N(%)	41,634 (14.8)	14,238 (15.9)	55,872 (15.1)
ASP, N(%)	30,024 (10.7)	20,584 (23.1)	50,608 (13.7)
Fruit&vegetable, N (%)	106,362 (37.8)	31,390 (35.2)	137,752 (37.2)
Vitamin, N(%)	42,615 (15.2)	12,120 (13.6)	54,735 (14.8)
Mineral, N(%)	61,513 (21.9)	17,344 (19.4)	78,857 (21.3)
Mean(SD) CRP, mg/L	2.09(3.94)	3.73(4.76)	2.49 (4.21)
**MetS components**			
Central obesity, N(%)	34,514 (12.28)	82,480 (92.37)	116,994 (31.59)
Dyslipidaemia for HDL, N(%)	3,6368 (12.94)	40,276 (45.11)	76,644 (20.70)
Dyslipidaemia for TG, N(%)	97,916 (34.84)	77,645 (86.96)	175,561 (47.41)
Hyperglycemia, N(%)	33,734 (12.01)	34,212 (38.31)	67,946 (18.35)
Hypertension, N(%)	184,781 (65.75)	82,905 (92.85)	267,686 (72.29)

*Abbreviations*: *MetS* metabolic syndrome, *IMD* Index of Multiple Deprivation, *HDL* high-density lipoprotein, *TG* triglyceride, *SBP* systolic blood pressure, *DBP* diastolic blood pressure, *BMI* body mass index, *MET* Metabolic equivalent of task, *Fruit&vegetable* Fruit&vegetable intake ≥ 5 portions per-day, *CRP* C-reactive protein

### Measurements

Our preliminary work described detailed measurement methods and quality control strategies [12, 13]. Briefly, all participants were invited to a physical examination centre for the collection of physical data and metabolic specimens. Waist circumference was measured twice consecutively at the level of the umbilicus using a skin ruler during calm breathing. Blood pressure was measured twice at 5-min intervals using an automated sphygmomanometer (HEM-7015IT; Omron, Kyoto, Japan) to minimize error. Blood specimens were drawn by trained physicians on a fasting basis, and meanwhile, blood glucose, HDL, triglyceride, and CRP concentrations were measured (Beckman Coulter (UK)). In addition, socio-demographic characteristics (including age, gender, ethnicity, Index of Multiple Deprivation), lifestyle (smoking, alcohol consumption, physical activity participation), medical history (diabetes, osteoarthritis, hypertension, surgical history), diet and medication (fruit and vegetable intake, dietary supplements, prescription drugs) were collected using a touchscreen questionnaire. Physical activity data were also assessed and categorized using adapted questions from the short International Physical Activity Questionnaire (IPAQ).

### Outcome ascertainment

Information on disease diagnoses in the UK Biobank database was categorized by professionals using ICD-10 codes and structured spreadsheets. We queried the database according to the ICD-10 codes for OA events registered in 2006–2021 and identified most OA events (excluding spinal OA, polyosteoarthritis of unknown origin, and other infectious OA, etc.). The diagnostic information primarily comes from primary care, hospital admission data, and self-report. Some participants have multiple instances of diagnostic information, but we used the first diagnosis as the outcome event. Hand OA (M18, M18.0, M18.1, M18.2, M18.3, M18.4, M18.5 and M18.9); Hip OA (M16, M16.0, M16.1, M16.2, M16.3, M16.4, M16.5, M16.6, M16.7 and M16.9); Knee OA (M17, M17.0, M17.1, M17.2, M17.3, M17.4, M17.5 and M17.9); Polyarthrosis (M15.1, M15.2). Participants were followed from initial recruitment until the first diagnosis of OA, death, loss to follow-up, or the end (December 31, 2021).

### Definition of MetS and its components

MetS and its components were defined and selected following the International Diabetes Federation (IDF) standards [11, 27]. Central obesity was defined according to waist circumference (≥ 94 cm in men or ≥ 80 cm in women). Hypertension was defined as systolic blood pressure (SBP) ≥ 130 mmHg and diastolic blood pressure (DBP) ≥ 85 mmHg or previously diagnosed or undergoing treatment for hypertension. Elevated triglycerides were defined as a plasma triglyceride level ≥ 1.7 mmol/L (150 mg/dL) or a prior diagnosis of elevated triglycerides or ongoing use of anti-triglyceride medication. Reduced HDL was defined as plasma HDL < 1.04 mmol/L (40 mg/dL) in men and plasma HDL < 1.29 mmol/L (50 mg/dL) in women; or being treated with various treatments for reduced HDL. Hyperglycemia was defined as fasting blood glucose ≥ 5.6 mmol/L (100 mg/dL) or a prior diagnosis of type 2 diabetes or ongoing treatment against type 2 diabetes. The above five symptoms are the MetS components. Also, central obesity plus any two or more components were defined as MetS.

### Statistical analysis

In the baseline characteristic description, categorical variables were expressed using percentages and frequencies, while continuous variables were presented using mean (standard deviation, SD) for normally distributed variables, and median (interquartile range) for skewed variables. Cox proportional risk models with age as the time variable were used to estimate the hazard ratio (HR) and 95% confidence interval (CI) of MetS and its components on the risk of OA. The proportional risk hypothesis was tested using the Schoenfeld residual method. All models were adjusted for age, and gender. In the basic model (model 1), we adjusted for baseline age and sex. In the lifestyle model (model 2), we further adjusted for body mass index (BMI), the Index of Multiple Deprivation (IMD), alcohol consumption, smoking, and physical activity. In the full model (model 3), further adjustments were made for non-steroidal anti-inflammatory drugs (NSAIDs), aspirin (ASP), vitamin, mineral and fruit & vegetable intake. In order to control for potential confounders, we adjusted for some lifestyle factors, including alcohol consumption (daily or almost daily, 1–4 times a week, 1–3 times a month, and special occasions only/never), smoking (current, previous and never), and fruit & vegetable intake (< 5 portions per day, ≥ 5 portions per day, or unknown/missing). To assess the association of MetS with OA risk in the inflammatory state, we stratified based on CRP levels. Based on previous studies of OA, we dichotomized serum CRP values for further analysis, i.e., low to moderate CRP (≤ 3 mg /L), and elevated CRP (> 3 mg /L) [28, 29]. We then defined four risk levels in relation to MetS: CRP < 3 mg/L with or without MetS and CRP ≥ 3 mg/L with or without MetS. The PRS of OA construction method can be found in Supplementary materials.

In addition, to check the robustness of the model and results, we performed extensive sensitivity analyses. First, to reduce the effects of selection bias and covariates, we used propensity scores in our preliminary analyses. We weighted each confounding factor and then proximity matched with a variable ratio one-to-many (1:2) within the caliper. Also, we set a caliper width of 0.2 standard deviations of the propensity scores on the logarithmic scale. Second, to evaluate the potential interaction of MetS with sociodemographic factors and lifestyle (including gender, age, alcohol consumption, smoking, drug and dietary supplement intake status), we performed subgroup analyses and fitted interaction terms with these factors in the model. Third, we then used restricted cubic spline (RCS) to reveal potential nonlinear associations between MetS components and OA risk in a fully adjusted model. We used a three-part model with three parts at the 10th, 50th, and 90th percentiles of each MetS component to flexibly model the association between each MetS factor and OA risk. Fourth, We estimated the number of MstS components and the cumulative incidence of OA by fitting Kaplan–Meier curves and compared them using the log-rank test. Fifth, to minimize reverse causality, we introduced a 4-year lag period for OA onset. In brief, participants who had an OA event four years after the start of follow-up were considered eligible. Finally, PRS were created following an additive model for previously published common genetic variants associated with OA. Then, this PRS was stratified into low (lowest quartile), intermediate (quartile 2–3) and high (highest quartile) risk based on values for all individuals (Supplementary materials).

We used the R software (version 3.5.0, R Foundation for Statistical Computing, Vienna, Austria) for all data analyses. All statistical tests were two-tailed, with p < 0.05 considered statistically significant.

## Results

### Baseline characteristics

A total of 370,311 eligible participants with a mean age of 56.45 years and a mean follow-up period of 11.79 years were included in this study. Table 1 summarizes the sociodemographic and pathological characteristics of these participants. Consistent with previous studies, participants with MetS had significantly higher blood pressure, fasting blood glucose, triglyceride levels, waist circumference, and IMD than participants without metabolic syndrome. Additionally, participants with MetS smoked more, engaged less in physical activity, and were more likely to have abnormal serum CRP levels. Interestingly, the MetS group did not show a trend toward greater alcohol consumption, and even lower than participants without MetS in terms of heavy alcohol consumption.

### Association between MetS and OA

**Fig. 1:**
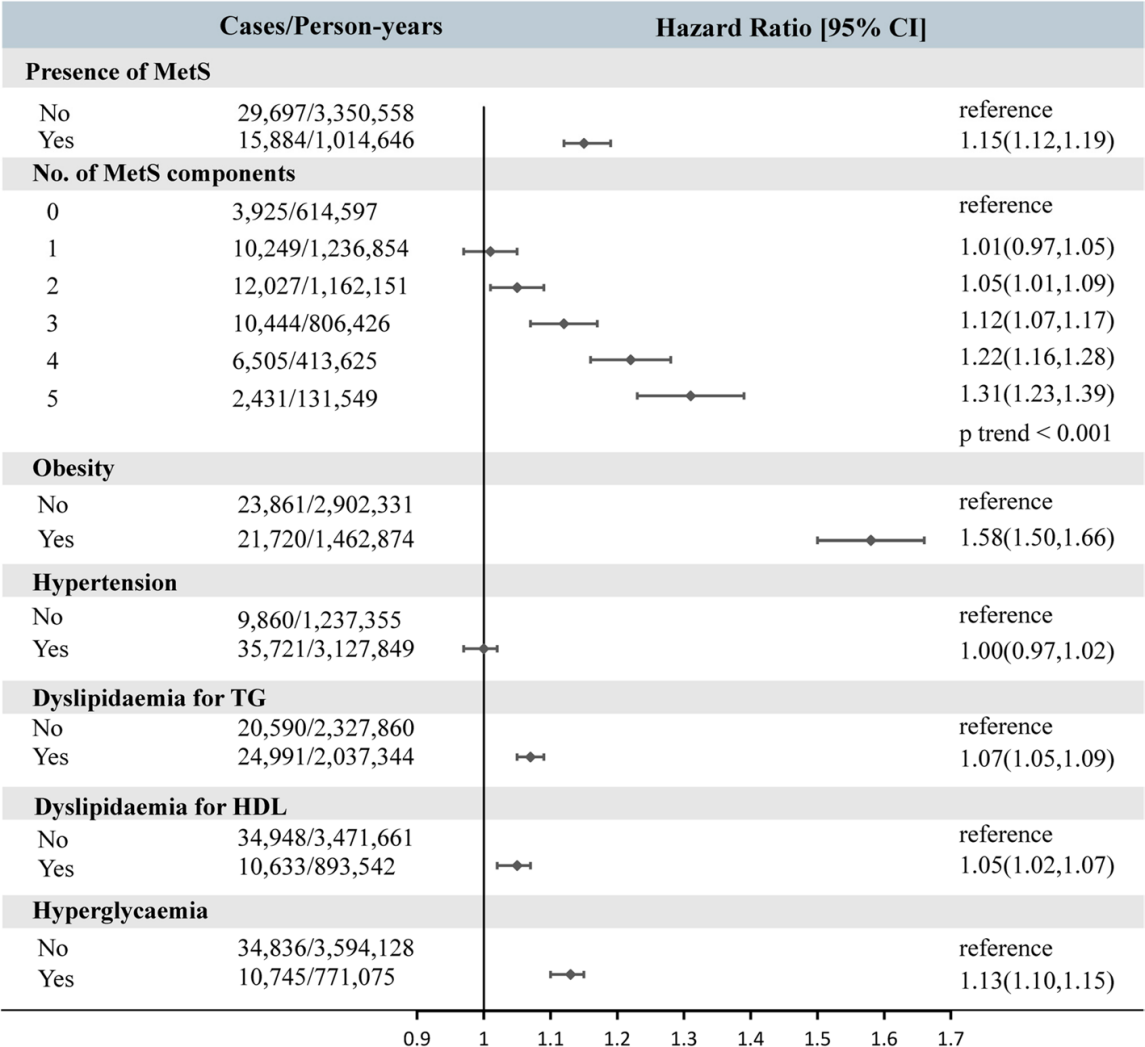
Risk of osteoarthritis according to metabolic syndrome and components. Models were adjusted for sex, age, BMI, IDM, alcohol consumption, smoking, physical activity, NSAIDs, ASP, vitamin, mineral and fruit & vegetable intake. Abbreviations:MetS, metabolic syndrome; HDL, high-density lipoprotein; TG, triglyceride. BMI, body mass index; IMD, Index of Multiple Deprivation

We recorded a total of 45,581 cases of OA over a median follow-up time of 12.48 years. The results showed that participants with MetS had a 19% increased risk of OA compared to those without MetS (HR = 1.19, 95% CI:1.16–1.23) (Supplementary 1). Even with a complete adjustment of lifestyle and other factors, participants with MetS still had a 15% higher risk of OA (HR = 1.15, 95%CI:1.12–1.19) (Fig. 1). After Propensity Score Matching, MetS remained associated with an increased risk of OA (HR = 1.17, 95% CI:1.14–1.21). The risk of OA could be significantly increased in patients with all five MetS components by 31% (HR = 1.31, 95%CI:1.23–1.39) compared to patients without any MetS components (Fig. 1).

**Fig. 2:**
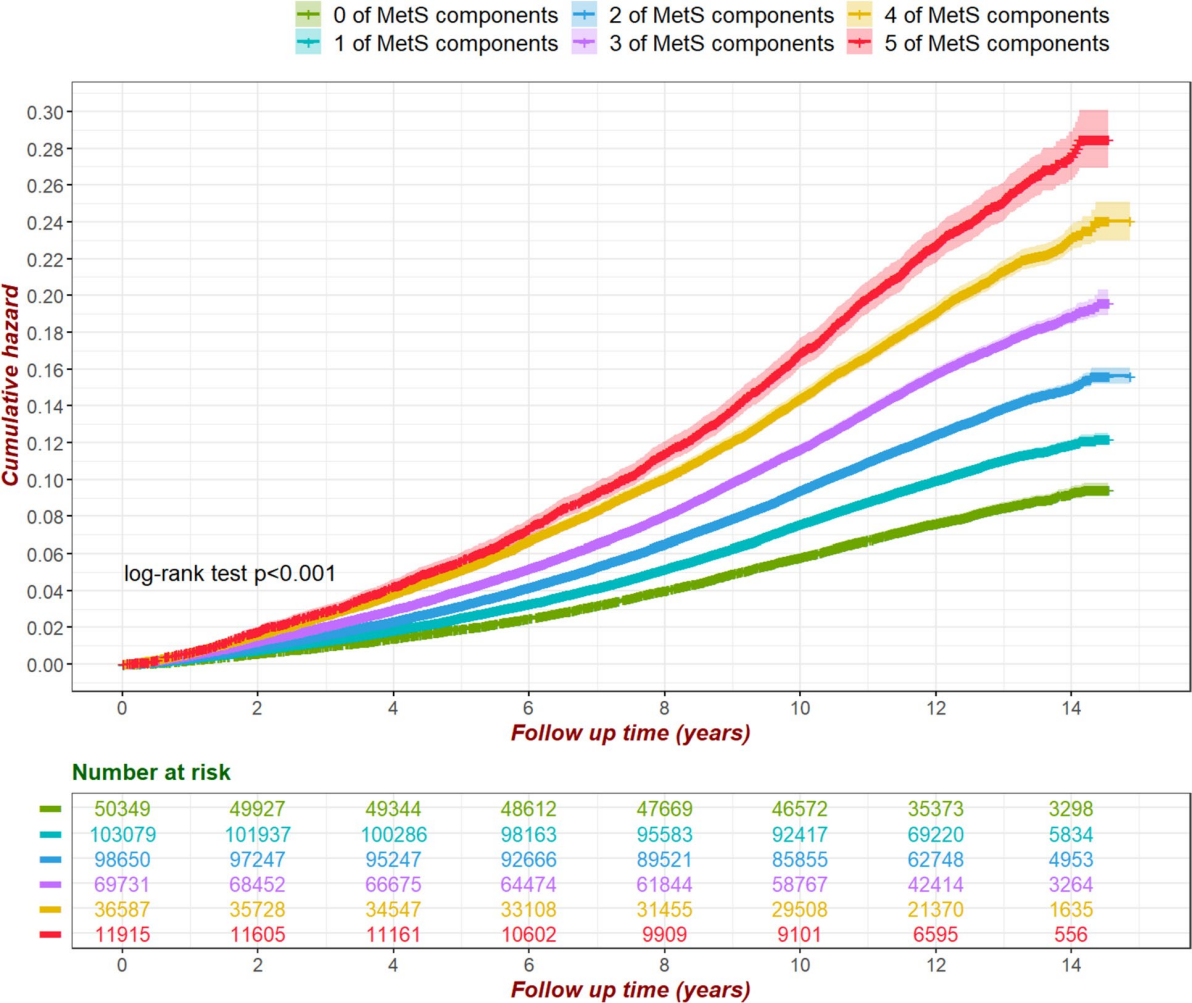
Kaplan–Meier curves showed the impact of MetS components on the risk of osteoarthritis, with significant differences between groups (log-rank test, *p* < 0.001). Models were adjusted for sex, age, BMI, IDM, alcohol consumption, smoking, physical activity, NSAIDs, ASP, vitamin, mineral and fruit & vegetable intake

**Fig. 3:**
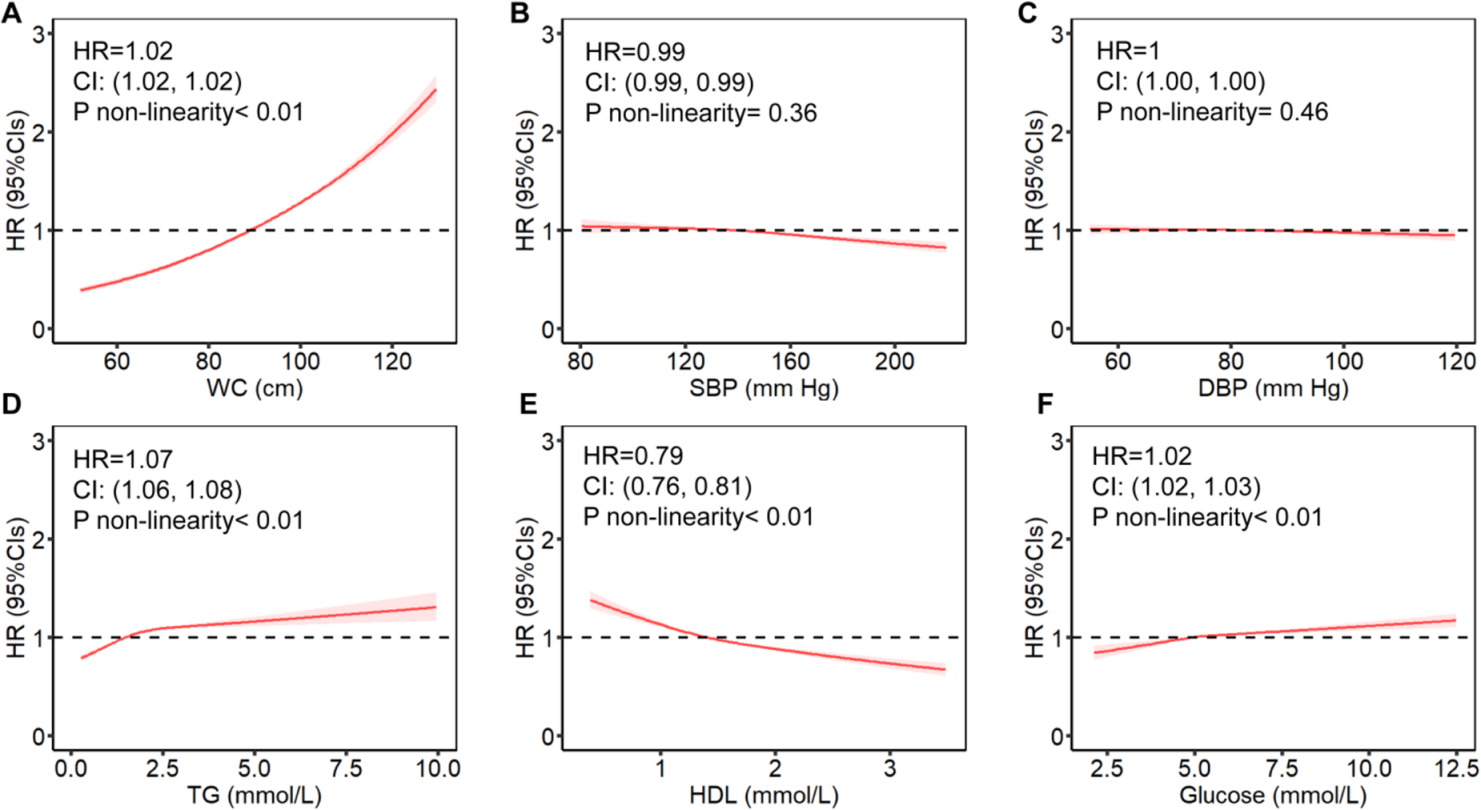
Estimated non-linear association between individual MetS component and the risk of osteoarthritis. **A **WC, **B **SBP, **C **DBP, **D **TG, **E **HDL, **F **Glucose. Models were adjusted for sex, age, BMI, IDM, alcohol consumption, smoking, physical activity, NSAIDs, ASP, vitamin, mineral and fruit & vegetable intake. Abbreviations: WC, Waist circumference; BMI, body mass index; HDL, high-density lipoprotein; TG, triglyceride; CRP, C-reactive protein; SBP, systolic blood pressure; DBP, diastolic blood pressure

The risk of OA tended to be increased significantly with the presence of MetS components. Kaplan–Meier curve analysis showed that having more MetS components was associated with a higher cumulative risk of OA, and the difference in risk between groups was statistically significant (*p* < 0.01) (Fig. 2). Among the MetS components, central obesity (HR = 1.58, 95%CI:1.5–1.66), hyperglycemia (HR = 1.13, 95%CI:1.1–1.15), dyslipidaemia in triglycerides (HR = 1.07, 95%CI:1.05–1.09) and HDL (HR = 1.05, 95%CI:1.02–1.07) were associated with OA risk with a positive correlation (*p* < 0.05). In addition, we assessed the potential nonlinear association of MetS components with OA risk (Fig. 3). The results showed that increased waist circumference (central obesity), elevated fasting glucose and triglycerides showed nonlinear associations with increased OA risk; while increased HDL was associated with decreased OA risk. Interestingly, although there was insufficient evidence that DBP was associated with OA risk, SBP beyond 150 mmHg appeared to be associated with a reduced risk of OA.

**Fig. 4:**
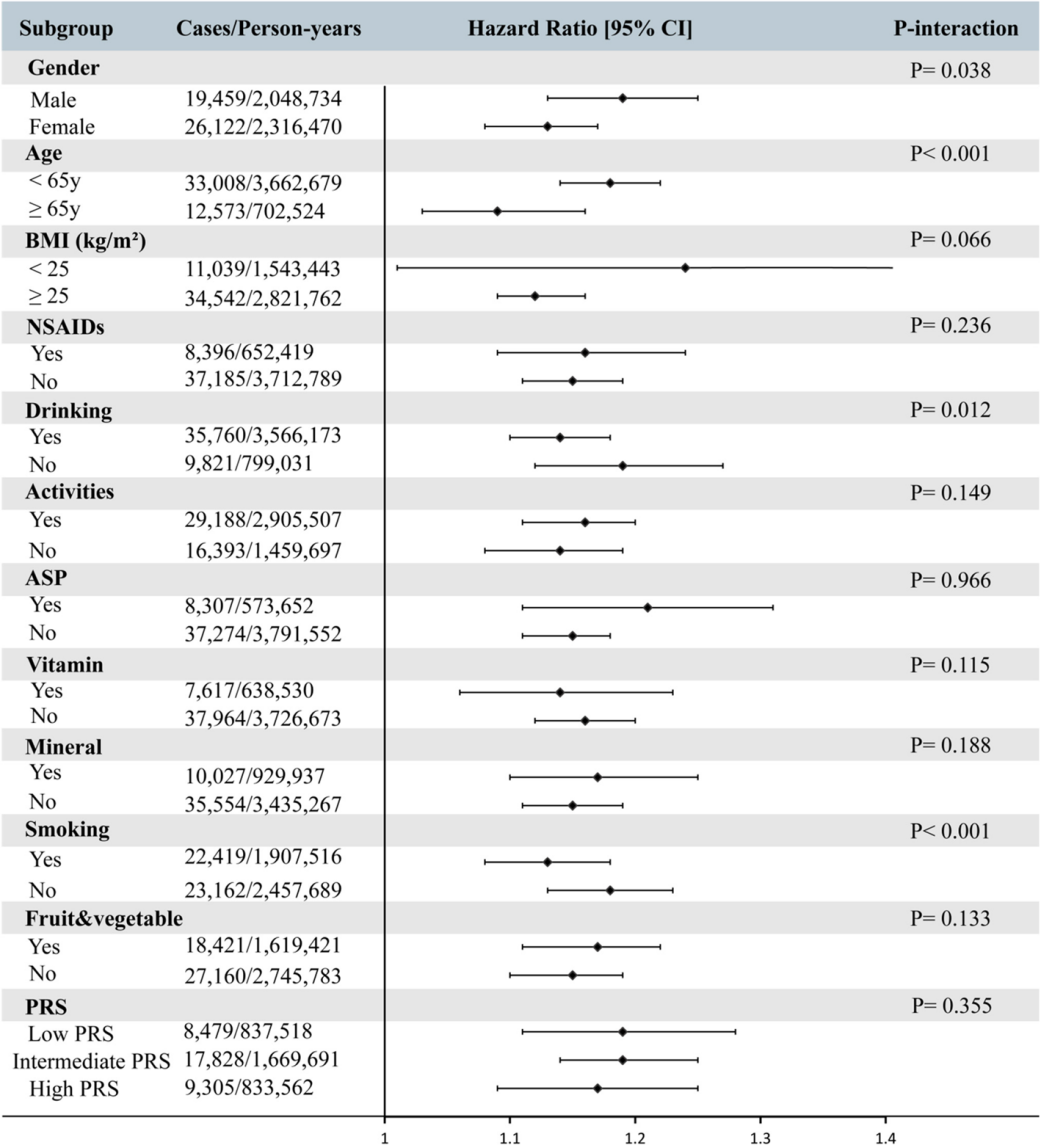
Subgroup analyses of MetS and risk of osteoarthritis. Abbreviations: MetS, metabolic syndrome

Next, we performed subgroup analysis and between-group interaction analysis. Our subgroup analysis revealed significant variations in the association between MetS and OA risk, particularly across gender, age, alcohol consumption, and smoking habits (Fig. 4). Specifically, MetS has a stronger association with OA risk among men and individuals under the age of 65. Interestingly, this association is also stronger among nonsmokers and nondrinkers. This could be attributed to the fact that these groups themselves have a higher risk of OA, thereby weakening the relative impact of MetS. Furthermore, the study did not find significant differences in this association among subgroups based on activity level, steroid and ASP consumption, vitamins, minerals, and vegetable and fruit intake. Although we observed that individuals with moderate and high PRS had a higher risk of developing OA compared to those with low PRS (supplementary 2), we did not find significant heterogeneity and interactions in the association between MetS and OA risk across these strata.

Additionally, when assessing the association between CRP and OA risk alone, the results suggested that elevated CRP (≥ 3 mg/L) was associated with an increased risk of OA (Table 2). With elevated CRP, MetS increased individuals' risk of OA by up to 35% (HR = 1.35, 95% CI:1.3–1.4).Finally, the results of sensitivity analyses showed that MetS was significantly associated with an increased risk of OA (HR = 1.15, 95% CI:1.11–1.19) even after a 4-year lag in exposure (Supplementary 3). Central obesity, triglyceride (TG) and hyperglycemia remained significantly associated with increased OA risk. These findings further demonstrates the robustness of our model and results.

**Table 2:** Risk of osteoarthritis according to CRP and the joint effect of MetS and CRP

	**No of cases/Person-years**	**Hazard Ratio (95% CI)**		
		Model 1	Model 2	Model 3
**CRP**				
< 3.0 mg/L	290,494/3,455,944	Reference	Reference	Reference
≥ 3.0 mg/L	79,817/909,260	1.23(1.21,1.26)	1.21(1.18,1.23)	1.20(1.16,1.23)
**CRP-Mets joint**				
No MetS/CRP < 3.0 mg/L	257,812/2,827,142	Reference	Reference	Reference
No MetS/CRP ≥ 3.0 mg/L	66,918/523,415	1.25(1.22,1.29)	1.22(1.19,1.26)	1.22(1.18,1.25)
MetS/CRP < 3.0 mg/dL	32,682/628,801	1.19(1.15,1.23)	1.16(1.13,1.21)	1.15(1.11,1.19)
MetS/CRP ≥ 3.0 mg/dL	12,899/385,844	1.42(1.37,1.47)	1.37(1.32,1.42)	1.35(1.30,1.40)

*Abbreviations*:*MetS* metabolic syndrome, *CRP* C-reactive protein

## Discussion

### Principal findings

We identified a total of 45,581 OA (prevalence 12.31%) in 370,311 participants with a median age of 58 years. This is similar to the results of Kuan et al.'s analysis based on English electronic health record (EHR) data, which showed a prevalence of 12.72% and a median age of 61 years [30]. With more than 12 years of follow-up, we found that MetS and its components were associated with an increased risk of OA. The more MetS components one had, the higher the risk of OA. The effect of MetS on the development of OA appeared to be greater in middle-aged adults under 65 years compared to older adults (≥ 65), which corroborates evidence from previous studies [31]. Furthermore, it is important to note that this association is independent of the genetic risk of OA. Meanwhile, elevated CRP is also associated with an increased risk of OA, and MetS strengthens this association. The present study validates the reliability of metabolic syndrome-associated OA epidemiologically.

### Comparison with other studies and possible explanations

In recent years, numerous clinical and observational studies have investigated the association between MetS, its components (such as obesity, hyperglycemia, hypertension, and hyperlipidemia) and the risk of OA [32, 33]. The definition of metabolic syndrome-associated OA well illustrates the high prevalence of OA in modern society, as the number of patients with MetS or with some of its components is exploding [34]. Our study confirmed this association and verified its robustness through multiple sensitivity analyses.

Both observational studies and clinical randomized trials have shown that obesity is an independent risk factor for OA [35–37]. Zeng et al. conducted a study using the UK Biobank cohort and found evidence supporting the association between increasing BMI from childhood to adulthood and an increased risk of OA [38]. Through multivariable regression analysis, we observed a significant link between obesity (central obesity) and OA risk. Due to limitations in observational studies, it is difficult to establish a causal relationship based on these findings. However, another study by Funck et al., also utilizing the UK Biobank, conducted MR analysis and confirmed a causal association between BMI and OA [39]. These results provide partial support for our research. Although the exact mechanism by which obesity increases the risk of OA is not fully understood, previous studies have shown that obesity not only increases mechanical stress on the joints but also alters the metabolism of serum cholesterol, triglycerides, and inflammatory factors, thereby increasing the risk of OA [40]. Wijesinghe et al. discovered, through single-cell RNA sequencing, that a specific subpopulation of fiber cells in the joint synovium of obese patients contributes significantly to the development of OA [41]. Animal studies have also confirmed that obesity is associated with a higher incidence of OA, and it appears that this effect may also be passed on to the next generation [42, 43].

Imbalances in lipid metabolism are not only a major cause of MetS (abnormal HDL, LDL and TG), but are also strongly associated with obesity. A prospective cohort study of women from the United Kingdom confirmed that hyperlipidemia is associated with the development of hand OA. Among them, HDL was inversely associated with the incidence of HOA, and TG increased the risk of developing HOA, but no association between LDL and HOA risk was observed [44]. Study from China also reported that hyperlipidemia was associated with knee pain and increased risk of KOA [45]. However, an MR analysis based on UK Biobank rejected the association between HDL, TG and OA risk, and noted that Apo and LDL were protective against OA [46]. In our study, we used restricted cubic spline analysis to confirm that HDL and TG were nonlinearly associated with OA risk. Specifically, elevated HDL was associated with a decreased risk of OA, but elevated TG increased the risk of OA. It has been proposed that macrophage infiltration and synovial inflammation result from disorders of lipid metabolism may be potential mechanism leading to OA [47].

The association between hyperglycemia/diabetes and OA is controversial. A portion of observational studies have found an association between hyperglycemia/diabetes and OA progression, but some studies have noted that this association is not reliable and is actually confounded by factors such as age and obesity [48–50]. In the present study, we found a significant association between hyperglycemia/diabetes mellitus and elevated risk of OA. To verify the robustness of this association, we adjusted for covariates including age, obesity, and gender in all models, and the results still showed a positive association between hyperglycemia/diabetes and elevated risk of OA (HR = 1.13, 95%CI:1.1–1.15). The proposed mechanism suggested that hyperglycemia may exacerbate OA progression through vascular factors, disruption of glucose metabolism or activation of pro-inflammatory mediators (cytokines, adipokines and reactive oxygen species) [51–53]. Moreover, cohort study and animal study have indicated that some first-line diabetes medications, such as metformin, may have a preventive effect on OA through glucose management, inhibition of inflammation, inhibition of oxidative stress, and regulation of autophagy [54, 55]. Although this effect needs further study, the risk of hyperglycemia/diabetes for OA is clear.

CRP has been reported not only to be an indicator of inflammation in OA and MetS, but also to promote both diseases [56, 57]. On the one hand, there is a consensus on CRP as a biomarker of systemic inflammation [58]. On the other hand, there is limited evidence that CRP, as a bioactive molecule, can contribute directly in multiple chronic diseases, including MetS and OA [18, 22, 24, 59, 60]. Perruccio et al. noted a dose–response relationship between CRP and OA symptoms, which may explain the inconsistent findings in the literature [61]. In our study, we identified that elevated CRP levels (≥ 3 mg/L) were associated with an increased risk of OA. And, we also found that the risk of OA could be significantly increased by 35% in the presence of elevated CRP in conjunction with MetS. Overall, these findings suggest that the association between MetS and OA risk is strengthened in the presence of elevated CRP and its indicated inflammatory state.

### Strengths and limitations of this study

The biggest strength of this study is the large cohort database and the complex research design, which is very close to real-world practice. Additionally, we performed several sensitivity analyses and quality controls to make our results more credible. The identification of the association between MetS and CRP for increased risk of OA complements the pathogenesis of OA. These findings could provide direct evidence for future mechanistic studies.

This study also has some limitations. First, Possible selection bias of "healthy volunteers" in the UK Biobank, which would limit the generalization of our findings. However, other comparable studies have suggested that the large size and heterogeneity of UK Biobank exposure measures provide valid scientific inferences of the association between exposure and outcome. Second, as the MetS components were measured only once, these risk factors may have changed during the follow-up, which is difficult to assess. However, our examination of repeated measures data (not shown) indicated that the change in these factors over time was not very significant. Third, the lack of detailed diagnostic information makes it difficult to know the severity of OA and assess its potential association with MetS. Finally, due to the nature of observational study design, causal associations are difficult to determine and only suggest associations.

## Conclusion

In summary, we provide evidence from an epidemiological study confirming that MetS and its components are positively associated with an increased risk of OA. Where obesity, hyperglycemia and elevated triglycerides were independently associated with OA risk. Furthermore, our findings suggest that MetS can more significantly increase the risk of OA in an inflammatory state. The specific mechanisms by which MetS and its components and CRP promote an increased risk of OA need further investigation to develop better OA prevention strategies.

### Supplementary Information

**Additional file 1: Supplementary 1.** Risk of osteoarthritis according to metabolic syndrome and components. **Supplementary 2.** Associations of genetic risk with OA. **Supplementary 3.** Sensitive analysis lag for 4 years.

## Data Availability

UK Biobank is an open-access resource, and the study website https://www.ukbiobank.ac.uk/ has information on available data and access procedures. Data sets used for the analysis will be made available under reasonable requests.
