# Supplementary material for: Metabolic syndrome increases osteoarthritis risk: findings from the UK Biobank prospective cohort study
Source: BMC Public Health. 2024 Jan 19;24:233. doi: 10.1186/s12889-024-17682-z (PMC10799367; doi:10.1186/s12889-024-17682-z)
Supplement: Supplementary file 1 — Additional file 1: Supplementary 1. Risk of osteoarthritis according to metabolic syndrome and components. Supplementary 2. Associations of genetic risk with OA. Supplementary 3. Sensitive analysis lag for 4 years. [file 12889_2024_17682_MOESM1_ESM.docx]

**Supplementary materials**

**Metabolic Syndrome Increases Osteoarthritis Risk: A Prospective Cohort Study**

**Method**:

**Polygenic risk score**: Polygenic risk scores were created following an additive model for previously published common genetic variants associated with OA.

1. The GWAS Data for all OA (including hip OA and knee OA) were obtained from the release version 9 of FinnGen ( <https://r9.risteys.finngen.fi/> )[1], and data of hand OA was obtained from the largest published GWAS of hand OA until now, with 303782 samples (case : 20901, control: 282881 )[2].
2. Quality control of GWAS datasets for PRS generation: For GWAS datasets of bone diseases, there are some quality control procedures conducted before the PRS calculation: (1) To match the UK Biobank data (build GRch37), GWAS datasets from the FinnGen were lifted over from reference genome build GRch38 to GRch37 using the hg38 to hg19 chain file downloaded from UCSC (<https://hgdownload.soe.ucsc.edu/goldenPath/hg19/liftOver/> ). (2) Variants with minor allele frequency (MAF) < 0.01 were excluded. (3) Indel variants were excluded. (4) Variants with alleles mismatched with the target genotyped data were excluded. (5) Ambiguous variants with palindromic alleles were excluded. (6) Variants with different effect alleles or strand with the target genotype data will be swapped or flipped. (7) Variants with duplicated names will only be kept at the first time it was observed. (8) Variants on the sex chromosomes were excluded because such chromosomes were unavailable in our UK Biobank genotype data.
3. Genotyping and imputation of UK Biobank data: Genotyping and imputation of UK Biobank data were conducted as the procedures published in previous study, in which the details of the array design, genotyping process and quality control can be found[3]. Subsequently, participants with a discrepancy between reported and genetic sex, sex chromosome aneuploidy, a high missingness or excess heterozygosity, participants of non-European ancestry, second-degree (or higher) related participants (kinship coefcient≥0.088) were excluded in our study. At last, among the 488377 participants with genetic data in the raw UK Biobank cohort, 378658 were remained for the following analysis.
4. Construction of PRS for OA: Based on variants extracted from corresponding GWAS after quality control procedures, we constructed polygenic risk scores for All OA in our study, using the pruning and thresholding approach (C+T). Firstly, SNPs were selected by a range of thresholds ( p < 1, 0.5, 5 × 10 ^-2^, 5 × 10 ^-3^, 5 × 10 ^-4^, 5 × 10 ^-5^, 5 × 10 ^-6^, 5 × 10 ^-7^, and 5 × 10 ^-8^ ). Meanwhile, SNPs were clumped with three linkage disequilibrium metric ( r2<0.1, 0.01, 0.001) within a distance of 1000kb, using the reference panel of European population from 1000 Genome project (<http://fileserve.mrcieu.ac.uk/ld/1kg.v3.tgz> )[4]. Next, PRS were created in an additive model, in which dosage of the effect alleles were summed weighted by effect sizes (the beta) . Therefore, up to 27 candidate PRS datasets were generated for each bone disease trait.
5. Selecting the best fitted PRS for OA: We then use logistic regression model to evaluate the association of every candidate PRS and OA in UK Biobank cohort. Considering the outcomes were binary variables, among the PRS that showed a significant association with the outcome ( *P* < 0.05 ), the one with the highest pseudo r-squared values (Nagelkerke’s ) was selected as the best-performing PRS for the following analysis.
6. Statistic analysis : The lift over analysis of the FinnGen data was conducted in R using Package ‘rtracklayer’ and ‘GenomicRanges’. Clumping of the base GWAS for PRS construction was performed using the PLINK 1.9 and generation of PRS was conducted on PLINK 2.0. Other procedures of quality control as well as selection of best fitted PRS were performed at R and python.

Then , this polygenic risk score was stratified into low (lowest quartile), intermediate (quartile 2–3) and high (highest quartile) risk based on values for all individuals.

**Reference**.

[1]. Kurki MI, Karjalainen J, Palta P, et al. FinnGen provides genetic insights from a well-phenotyped isolated population [published correction appears in Nature. 2023 Feb 24;:]. *Nature*. 2023;613(7944):508-518. doi:10.1038/s41586-022-05473-8

[2]. Boer CG, Hatzikotoulas K, Southam L, et al. Deciphering osteoarthritis genetics across 826,690 individuals from 9 populations. *Cell*. 2021;184(24):6003-6005. doi:10.1016/j.cell.2021.11.003

[3]. Bycroft C, Freeman C, Petkova D, et al. The UK Biobank resource with deep phenotyping and genomic data. *Nature*. 2018;562(7726):203-209. doi:10.1038/s41586-018-0579-z

[4]. 1000 Genomes Project Consortium, Abecasis GR, Altshuler D, et al. A map of human genome variation from population-scale sequencing [published correction appears in Nature. 2011 May 26;473(7348):544. Xue, Yali [added]; Cartwright, Reed A [added]; Altshuler, David L [corrected to Altshuler, David]; Kebbel, Andrew [corrected to Keebler, Jonathan]; Koko-Gonzales, Paula [corrected to Kokko-Gonzales, Paula]; Nickerson, Debbie A [corrected to Nickerson, Debo]. *Nature*. 2010;467(7319):1061-1073. doi:10.1038/nature09534.

**Supplementary 1. Risk of osteoarthritis according to metabolic syndrome and components.**

|  | **No of cases/**  **Person-years** | **Hazard Ratio (95% CI)** | | |
| --- | --- | --- | --- | --- |
|  |  | Model 1 | Model 2 | Model 3 |
| **Presence of MetS** |  |  |  |  |
| No | 29,697/3,350,558 | Reference | Reference | Reference |
| Yes | 15,884/1,014,646 | 1.19(1.16,1.23) | 1.16(1.13,1.20) | 1.15(1.12,1.19) |
| **No. of MetS components** |  |  |  |  |
| 0 | 3,925/614,597 | Reference | Reference | Reference |
| 1 | 10,249/1,236,854 | 1.02(0.98,1.05) | 1.00(0.97,1.04) | 1.01(0.97,1.05) |
| 2 | 12,027/1,162,151 | 1.07(1.03,1.11) | 1.05(1.01,1.09) | 1.05(1.01,1.09) |
| 3 | 10,444/806,426 | 1.15(1.11,1.20) | 1.13(1.08,1.18) | 1.12(1.07,1.17) |
| 4 | 6,505/413,625 | 1.27(1.21,1.33) | 1.23(1.17,1.29) | 1.22(1.16,1.28) |
| 5 | 2,431/131,549 | 1.41(1.33,1.49) | 1.32(1.24,1.40) | 1.31(1.23,1.39) |
|  |  | *P* trend < 0.001 | *P* trend < 0.001 | *P* trend < 0.001 |
| **Central Obesity** |  |  |  |  |
| No | 23,861/2,902,331 | Reference | Reference | Reference |
| Yes | 21,720/1,462,874 | 1.65(1.56,1.73) | 1.60(1.52,1.69) | 1.58(1.50,1.66) |
| **Hypertension** |  |  |  |  |
| No | 9,860/1,237,355 | Reference | Reference | Reference |
| Yes | 35,721/3,127,849 | 1.00(0.98,1.02) | 1.00(0.97,1.02) | 1.00(0.97,1.02) |
| **Dyslipidaemia for TG** |  |  |  |  |
| No | 20,590/2,327,860 | Reference | Reference | Reference |
| Yes | 24,991/2,037,344 | 1.09(1.07,1.11) | 1.08(1.05,1.10) | 1.07(1.05,1.09) |
| **Dyslipidaemia for HDL** |  |  |  |  |
| No | 34,948/3,471,661 | Reference | Reference | Reference |
| Yes | 10,633/893,542 | 1.08(1.06,1.11) | 1.06(1.03,1.08) | 1.05(1.02,1.07) |
| **Hyperglycaemia** |  |  |  |  |
| No | 34,836/3,594,128 | Reference | Reference | Reference |
| Yes | 10,745/771,075 | 1.16(1.13,1.18) | 1.13(1.11,1.16) | 1.13(1.10,1.15) |

**Model 1**: basic model only adjusted for age, gender;

**Model 2**: additionally adjusted BMI, IDM, Drinking, activity, and smoking;

**Model 3**: additionally adjusted for NSAIDS, ASP, Fruit&vegetable, vitamin, and mineral intake.

**Supplementary 2. Associations of genetic risk with OA.**

| PRS | N | Cases | HR (95% CI) | P-value |
| --- | --- | --- | --- | --- |
| Low PRS | 70841 | 8,479 | reference |  |
| Intermediate PRS | 141682 | 17,828 | 1.05 (1.03,1.08) | < 0.01 |
| High PRS | 70841 | 9,305 | 1.10 (1.07,1.13) | < 0.01 |

**Supplementary 3. Sensitive analysis lag for 4 years.**


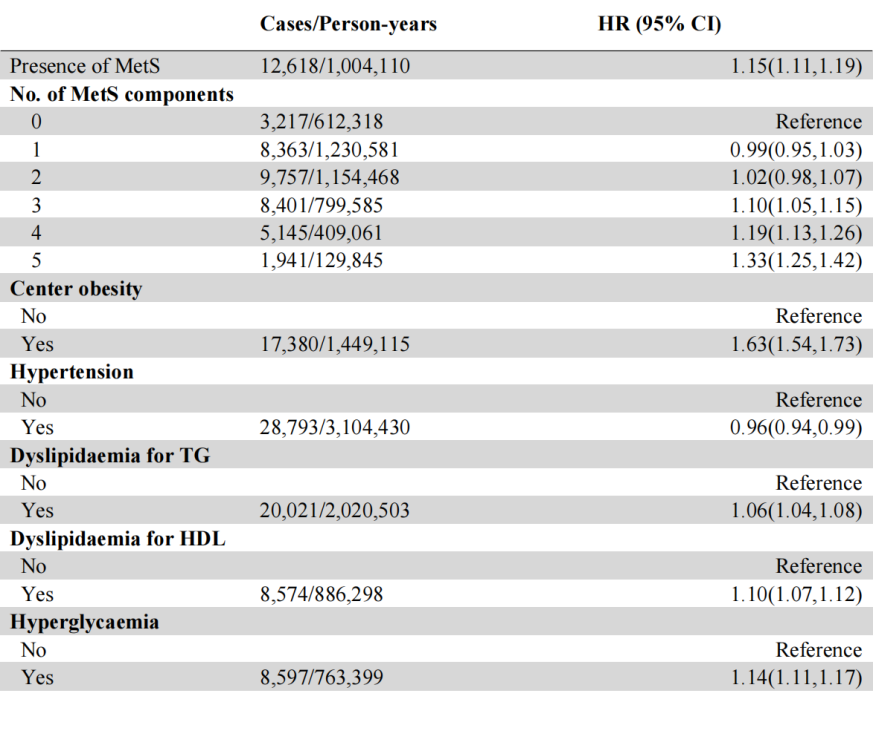

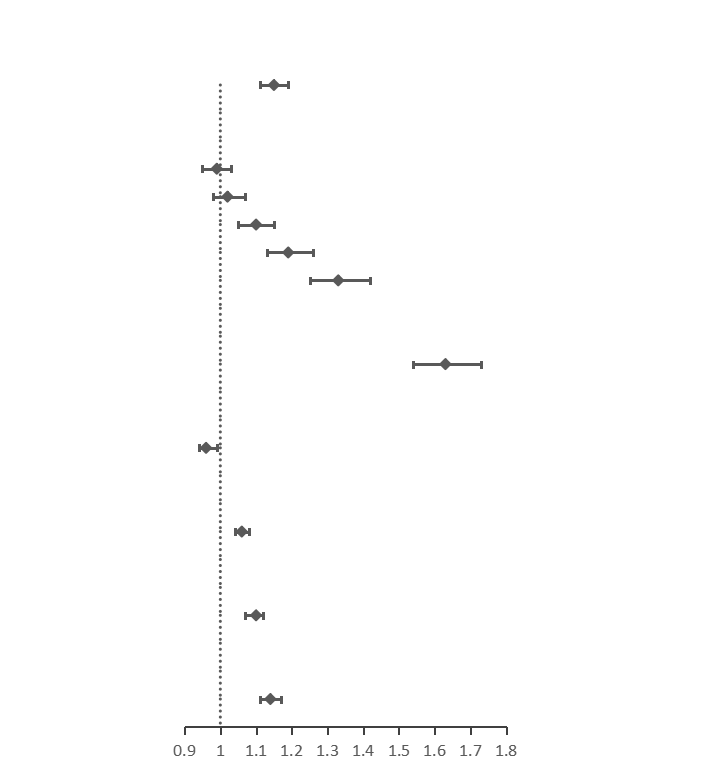


**Abbreviations**: MetS, metabolic syndrome; HDL, high-density lipoprotein; TG, triglyceride; BMI, body mass index.
